# Symptom structure of complex posttraumatic stress disorder among Chinese young adults with childhood trauma: a network analysis

**DOI:** 10.1186/s12888-023-05423-2

**Published:** 2023-12-05

**Authors:** Luxi Yang, Chenguang Wei, Yiming Liang

**Affiliations:** Shanghai Key Laboratory of Mental Health and Psychological Crisis Intervention, School of Psychology and Cognitive Science, East China Normal University, Shanghai, 200062 China

**Keywords:** Complex posttraumatic stress disorder, Network analysis, China, Bayesian network

## Abstract

**Background:**

The 11th revision of the World Health Organization’s International Classification of Diseases (ICD-11) includes a new disorder, complex posttraumatic stress disorder (CPTSD), the diagnostic applicability of which has not been discussed sufficiently in Chinese culture. The network approach to psychopathology enables investigation of the structure of disorders at the symptom level, which allows for analysis of direct symptom interactions. The main objectives of the present study were to explore CPTSD symptom structure and identify key symptoms in CPTSD among young adults in China.

**Methods:**

The present study collected a large, stratified sample of Beijing university students (1368), ranging from 18 to 25 years old, the majority of whom (65.4%) were female. CPTSD symptoms were assessed using the International Trauma Questionnaire (ITQ). A regularized partial correlation network and Bayesian network were applied to estimate the network structure and the upstream symptoms of CPTSD, respectively.

**Results:**

The regularized partial correlation network showed that the high central symptoms were feelings of failure and hypervigilance, while the bridge symptom between posttraumatic stress disorder (PTSD) and disturbance in self-organization (DSO) domains was long-term upset. The Bayesian network showed that external avoidance and hypervigilance symptoms were upstream in CPTSD symptoms.

**Conclusions:**

Hypervigilance is a central symptom that can be predictive of other symptoms of CPTSD. While feeling of failure is also a highly central symptom, it may be influenced by other symptoms. In the diagnosis and intervention of CPTSD, more attention should be given to hypervigilance symptoms.

**Supplementary Information:**

The online version contains supplementary material available at 10.1186/s12888-023-05423-2.

## Introduction

Although under discussion over the years, complex posttraumatic stress disorder (CPTSD) has recently been officially recognized as a distinct psychiatric disorder by the World Health Organization (WHO). In 2018, CPTSD was encompassed in the 11th revision of the International Classification of Diseases (ICD-11) as a sibling disorder to posttraumatic stress disorder (PTSD) [1]. Both CPTSD and PTSD are listed in the diagnostic criteria of trauma-related mental disorders of the ICD-11. Cases with PTSD symptoms have reexperience of trauma, avoidance of trauma cues and a sense of threat, while cases with CPTSD have symptoms of disturbance in self-organization (DSO) in addition to symptoms of PTSD. DSO symptoms include affective dysregulation, negative self-concept and difficulties in relationships. In other words, in the ICD-11, patients who only meet the diagnostic criteria for PTSD are diagnosed with PTSD, while patients who meet the diagnostic criteria for both PTSD and DSO are diagnosed with CPTSD [1]. Previous studies have shown that CPTSD has close associations with prolonged, repeated and especially human-induced trauma, such as childhood abuse, domestic violence, and sexual assault [2, 3]. However, PTSD (not accompanied by DSO symptoms) has strong associations with sudden and major traumatic events (i.e., natural disasters, accidents, etc.).

A great number of factor analytic studies have been investigating the latent structure of CPTSD for years [4–6]. A systematic review of such studies by Redican and colleagues supported the concept of capturing the structure of CPTSD with two models: a correlated six-factor model (re-experiencing, avoidance, threat, affect dysregulation, negative self-concept, and disturbed relationships) and a two-factor second-order model (PTSD and DSO), which demonstrated the diagnostic criteria in ICD-11 [7]. Most previous studies found that the two-factor second-order model was the best fit among clinical samples, while the correlated six-factor first-order model was the best fitting model among community studies [7].

The network approach is often used in research involving assessment of psychopathological structure [8, 9]. The network approach defines mental disorders as a series of interacting symptoms [10]; each node represents a specific symptom, and edges represent relationships between symptoms in a network. The triggering of one symptom may lead to the activation of other symptoms. In a network study, central symptoms can also be revealed, which are most closely connected with other symptoms and may activate other symptoms [11]. The relationship between two different mental disorders or two subgroups within one mental disorder is recognized through bridge symptoms [12], which is also of great interest of CPTSD research, since this disorder has two symptom clusters – PTSD and DSO.

Six studies to date have explored symptom networks in CPTSD using the network approach [13–18], and five of them used community samples [13–17]. Levin and colleagues identified “feelings of worthlessness” as the most central symptom [16]. This remarkable finding was proven repeatedly by other CPTSD studies in Germany, Israel, the UK, and the USA, which found “feelings of worthlessness” to be the most central symptom [14], and the same result was found for samples in Austria, the United Kingdom and Lithuania [15]. Karatzias and colleagues compared network structures across different trauma types and found that negative self-concept was particularly central for the poly-traumatized group [15].

Traumatic events that trigger CPTSD are closely related to society and culture. Thus, the cross-cultural consistency and specificity are extremely critical for CPTSD’s diagnostic structure. The six studies mentioned above were conducted in various areas or countries [13–18]. However, no studies have explored CPTSD networks in Asian cultures.

Central symptoms have clinical implications. Bridge symptoms can inform clinicians about the potential connections between two symptom clusters. However, the bridge strength index has long been ignored in the literature on CPTSD networks. Further, limited by cross-sectional data, all the connections between symptoms in the previous studies are nondirectional. Recently, researchers have considered Bayesian network analysis as a method for determining symptom activation order using cross-sectional data, which estimates the upstream and downstream order of symptoms by generating a directed acyclic graph [11].

The current study examined the structure of the CPTSD symptom network in a large population of young Chinese adults who had traumatic experiences before 18 years old. We focused on the central symptoms and bridge symptoms, attempting to determine if the core symptoms are consistent with those in other cultures. Furthermore, we employed Bayesian networks to estimate the direction of interaction between symptoms.

## Methods

### Participants

Participants in this study consisted of students attending universities in Beijing, China. Sixty-seven universities in Beijing were divided into 13 types according to their disciplines. Taking the running levels of the universities into account as well, in other words, trying to cover not only key universities but also ordinary universities, we included 31 universities in this study: comprehensive (5), science (5), engineering (5), agriculture (2), normal (2), finance and economics (3), forestry (1), politics and law (1), medicine (1), language (3), nationality (1), art (1) and sports (1). Random stratified sampling strata were made on the universities, majors (liberal arts or sciences) and grades. In this way, we ensured the diversity and representativeness of the participants. Participants first read the instructions for the present study. Those who agreed to participate provided informed consent. Then, they were given an online questionnaire and completed the assessment. The distribution and collection of questionnaires were conducted by teachers in the universities. We continuously recruited students until the number in each stratum reached the number we formulated in advance. Overall, 2048 participants from 29 universities completed the survey. We first screened to obtain valid data, and 221 participants were excluded due to careless answers (e.g., failure to pass the attention check items or answering the same answer to each item). Then, 1827 (89.2%) valid data points were further screened according to the inclusion criteria in this study: (a) had direct or indirect trauma histories, which were determined by the score of the Life Events Checklist for DSM-5; and (b) were aged 18–25 years when the data were collected. Finally, 1368 met the inclusion criteria.

Approval for this study was granted by the ethics committee on human research protection of East China Normal University. All participants gave informed consent. They had a mean age of 20.36 ± 1.45 years, and there were more women (65.4%) than men. Other demographics were the prevalence of PTSD and CPTSD, the participants’ majors and the education levels of their parents (see Table 1).

**Table 1:** Demographics information (*n* = 1368)

Items		*n* (%)
Gender, *n*_women_(%)		895 (65.4)
Prevalence of PTSD, *n*_ptsd_(%)		70 (5.1)
Prevalence of CPTSD, *n*_cptsd_(%)		119 (8.7)
Major,	Philosophy	9 (0.7)
	Economics	125 (9.1)
	Law	138 (10.1)
	Education	45 (3.3)
	Literature	112 (8.2)
	History	6 (0.4)
	Science	281 (20.5)
	Engineering	317 (23.2)
	Agriculture	48 (3.5)
	Medical science	59 (4.3)
	Management	139 (10.2)
	Art	15 (1.1)
	Military	0 (0)
	Others	74 (5.4)
Education level of father, *n* (%)	Primary school	95 (6.9)
	Junior middle school	246 (18.0)
	Senior middle school	412 (30.1)
	College	530 (38.7)
	Postgraduate	75 (5.5)
	Others	10 (0.7)
Education level of mother, *n* (%)	Primary school	129 (9.4)
	Junior middle school	268 (19.6)
	Senior middle school	438 (32.0)
	College	478 (34.9)
	Postgraduate	47 (3.4)
	Others	8 (0.6)

### Measurement

#### Trauma history

To classify childhood traumatic events that may contribute to CPTSD symptoms, we used a revised version of the Life Events Checklist for DSM-5 (LEC-5), which has 17 items such as natural disasters, physical or sexual assault, serious injury, and violent death (homicide or suicide) [19]. Considering that the target population were young Chinese adults, four events that would hardly happen to them were deleted: exposure to war, captivity, serious accident at work and severe human suffering. For each event, participants were asked to recall and indicate the type of exposure (e.g., whether they directly experienced or witnessed the event and whether it was related to occupational activities) before they were 18 years old. Each item was scored on a six-point Likert scale, ranging from 0 (does not apply) to 5 (happened to me). Only those young adults who reported having witnessed or experienced at least one event were considered to have childhood traumatic experience and were identified as having a history of childhood trauma.

#### CPTSD symptoms

The International Trauma Questionnaire (ITQ) was adopted to measure ICD-11 PTSD and CPTSD [20]. The Chinese version of ITQ was utilized to assess CPTSD symptoms in this study [21]. The ITQ consists of 18 items, 12 of which correspond to 12 symptoms of CPTSD and 6 that measure functional impairment. PTSD symptoms (re-experiencing, avoidance, and sense of current threat) were assessed by six items, with each symptom measured by two items. There were three items assessing functional impairment associated with PTSD symptoms. Similarly, DSO symptoms (negative self-concept, affective dysregulation, and disturbances in relationship) were assessed by six symptom-related items and three function-related items. All items were rated on a 5-point Likert scale ranging from 0 (not at all) to 4 (extremely). The diagnosis of PTSD or DSO requires all three PTSD or DSO symptoms to be present (scored 2 or greater), while functional impairment was also observed (at least one of the three function-related items scored 2 or greater). CPTSD was diagnosed when both PTSD and DSO met the criteria. In other words, participants who only meet the diagnostic criteria for PTSD are diagnosed with PTSD, while participants who meet the diagnostic criteria for both PTSD and DSO are diagnosed with CPTSD. In this study, Cronbach’s alpha of the scale was 0.88.

### Data analysis

We relied on IBM SPSS Statistics 23.0 to evaluate the prevalence of the reported childhood traumatic events and descriptive statistics of CPTSD symptoms. There was no missing data.

#### Confirmatory factor analysis

**Fig. 1:**
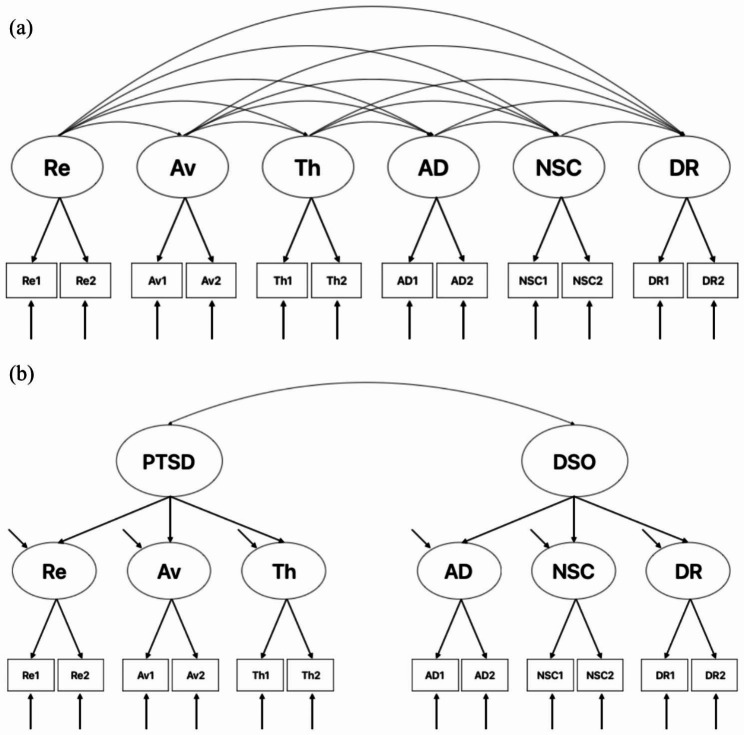
Correlated six-factor first-order model (**a**) and two-factor second-order model (**b**). *Notes*: Re = re-experiencing; Av = avoidance; Th = sense of threat; AD = affective dysregulation; NSC = negative self-concept; DR = disturbed Relationships; Re1: nightmares; Re2: flashbacks; Av1: internal avoidance; Av2: external avoidance; Th1: hypervigilance; Th2: exaggerated startle response; AD1: long-term upset; AD2: emotional numbing; NSC1: feelings of failure; NSC2: feelings of worthlessness; DR1: feeling distant or cut off from others; DR2: difficulties feeling close to others

We first tested the factor structure of ITQ in our sample. The correlated six-factor first-order model (model 1) and the two-factor second-order model (model 2; see Fig. 1) were tested using confirmatory factor analysis (CFA). The CFA analyses were performed in Mplus 8.3 [22]. We evaluated the model fit using the following fit indices: chi-squared test, the comparative fit index (CFI) [23], the Tucker‒Lewis index (TLI) [24], and the root mean square error of approximation (RMSEA) [25]. CFI and TLI values ≥ 0.95 reflect excellent model fit; RMSEA values ≤ 0.08 and ≤ 0.06 reflect acceptable and excellent model fit, respectively. The change in the RMSEA value (ΔRMSEA) was used to compare the two CFA models, and a ΔRMSEA value of ≥ 0.015 suggests a meaningful difference in model fit [26].

#### Regularized partial correlation network

A statistical procedure described by Epskamp and Fried was conducted to identify the overall network of ICD-11 CPTSD symptoms [27]. All analyses were performed using R 4.1.2 and visualized with the R package *qgraph* [28]. Because previous studies found that CPTSD is more likely associated with repeated trauma and poly-traumatized exposure [13], we first performed network analysis in all samples with trauma history and then in people who experienced 2 or more trauma types. Finally, we compared the results of network analysis in two samples.

The partial correlation network was used to prescribe the association parameters between all nodes according to Gaussian graphical models (GGMs). Sixty-six pairwise associated parameters between a total of 12 symptom nodes were estimated using the least absolute shrinkage and selection operator (LASSO) [29].

Centrality estimation was made for every symptom in the network, consisting of two categories of indices: strength centrality and bridge strength. Strength centrality, the most common and stable centrality metric [30], refers to the weighted sum of all edges connected to a particular node [31]. It was analyzed to predict the most connected node in a network. Bridge strength indicates a node’s total connectivity with other disorders or other clusters in the same disorder [12]. It was obtained for the two distinct subgroups of CPTSD (PTSD and DSO).

Robustness analyses were performed by the R package bootnet [32]. To account for the edge weight accuracy, we used the R package *bootnet* to bootstrap the 95% confidence intervals (CIs) around the edge weights (bootstrapped samples = 1000). Fewer overlaps among those CIs indicate higher accuracy. Centrality stability was estimated by case-dropping bootstraps, which extracted subsets from the original data, calculated node centrality based on the subsets, and correlated the ranking results of subset centrality with that of the total sample. The correlation-stability coefficients (CS coefficients) were used as an outcome measure. When it is above 0.50, it indicates that the stability is strong [33]. The edge weight difference test and centrality difference test were also estimated.

#### Bayesian network

A Bayesian network was explored, with accessible causal interpretations of relationships between nodes [11]. We used the hill-climbing algorithm [34] provided in the R package *bnlearn* [35] to evaluate the directed edges (i.e., arrows) among symptoms, with all variables placed in a putative causal cascade, where upstream variables constitute the cause of downstream variables. The modeling process randomly added, subtracted and reversed the direction of edges while gradually optimizing the Bayesian information criterion (BIC) at the same time. For the stability of the Bayesian network, multiple bootstrapping samples were drawn, and their averaged results were used as the final network [36]. The Bayesian network was visualized in the form of a directed acyclic graph (DAG), and the source nodes or the upstream nodes revealed the most noteworthy symptoms in the Bayesian network.

## Results

### Descriptive statistics

The prevalence of reported childhood traumatic events is shown in Table 2. Physical assault was the most prevalent traumatic event (54.8%). Among 1368 participants who reported childhood traumatic events, 528 (38.6%) participants reported having experienced a single traumatic event, 608 (44.4%) participants reported exposure between 2 and 4 traumatic events, 216 (15.8%) participants reported exposure between 5 and 8 traumatic events, and 16 (1.2%) participants reported exposure more than 8. The most impactful traumatic experience occurring over time is shown in Table 2. The majority (41.2%) reported that the most impactful trauma occurred between the past one to five years. The mean scores, standard deviations and prevalence of CPTSD symptom severity ratings are shown in Table 3.

**Table 2:** Information on the reported childhood traumatic events (*n* = 1368)

	Respondents (*n*)	Percentage of the sample (%)
**Event**		
Physical assault	749	54.8
Transportation accident	700	51.2
Natural disasters	573	41.9
Life-threatening illness or injury	493	36.0
Sudden accidental death to a loved one	383	28.0
Unwanted or uncomfortable sexual experience	359	26.2
Causing serious injury or death to someone else	318	23.2
Assault with a weapon	295	21.6
Fire or explosion	287	21.0
Sudden death to a loved one	199	14.5
Exposure to toxic substance	96	7.0
Sexual assault	70	5.1
**The most impactful trauma occurred time**		
With in 6 months	234	17.11
6 months-12 months	161	11.77
1 year-5 years	563	41.15
5 years-10 years	264	19.30
More than 10 years	146	10.67

**Table 3:** Mean scores, standard deviations and prevalence of CPTSD symptoms

Symptom	Short code	*M*	*SD*	Percentage (%)
Nightmares	RE1	1.01	1.12	26.5
Flashbacks	RE2	1.15	1.19	33.7
Internal avoidance	AV1	1.41	1.28	41.0
External avoidance	AV2	1.39	1.31	40.8
Hypervigilance	TH1	0.97	1.21	28.8
Exaggerated startle response	TH2	0.99	1.19	28.9
Long-term upset	AD1	1.54	1.08	45.3
Emotional numbing	AD2	1.19	1.19	34.7
Feelings of failure	NSC1	1.16	1.21	33.3
Feelings of worthlessness	NSC2	0.92	1.18	24.7
Feeling distant or cut off from others	DR1	0.93	1.14	26.8
Difficulties feeling close to others	DR2	1.34	1.29	38.4

### CFA results

The CFA results are reported in Table 4. Both models 1 and 2 showed excellent fit for the TLI and CFI values and acceptable fit for the RMSEA value. Compared to model 2, model 1 had better fit indices in TLI, CFI and RMSEA values, but a ΔRMSEA value of 0.003 did not suggest a meaningful difference in model fit between the 2 models.

**Table 4:** Fit indices for CFA of two models

Model	χ^2^	*df*	χ^2^/*df*	RMSEA	CFI	TLI
Model 1	254.324	39	6.521	0.064	0.976	0.959
Model 2	339.646	47	7.227	0.067	0.967	0.954

*Note*: Model 1 = the correlated six-factor first-order model; Model 2 = the two-factor second-order model; CFI = comparative fit indices; TLI = Tucker Lewis indices; RMSEA = root mean square of approximation.

### Regularized partial correlation network

**Fig. 2:**
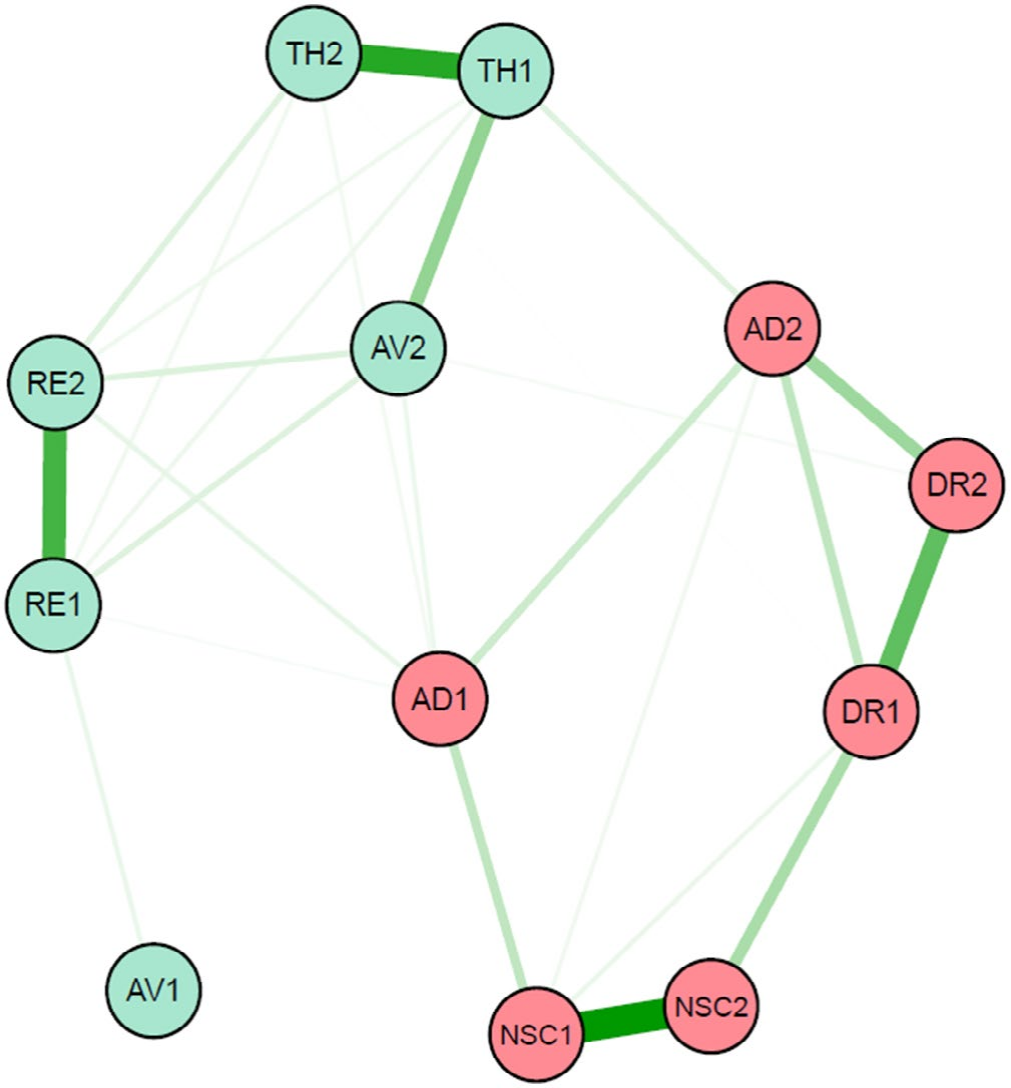
Regularized partial correlation network of CPTSD symptoms. *Notes*: RE1: nightmares; RE2: flashbacks; AV1: internal avoidance; AV2: external avoidance; TH1: hypervigilance; TH2: exaggerated startle response; AD1: long-term upset; AD2: emotional numbing; NSC1: feelings of failure; NSC2: feelings of worthlessness; DR1: feeling distant or cut off from others; DR2: difficulties feeling close to others

Figure 2 depicts the results of the regularized partial correlation network analysis in 1368 participants with childhood trauma. The connections of symptoms within the same cluster were strong (e.g., TH1:TH2; NSC1:NSC2). At the same time, there are also some symptom associations between PTSD and DSO (e.g., AD1:AV2; AD1:RE2).

**Fig. 3:**
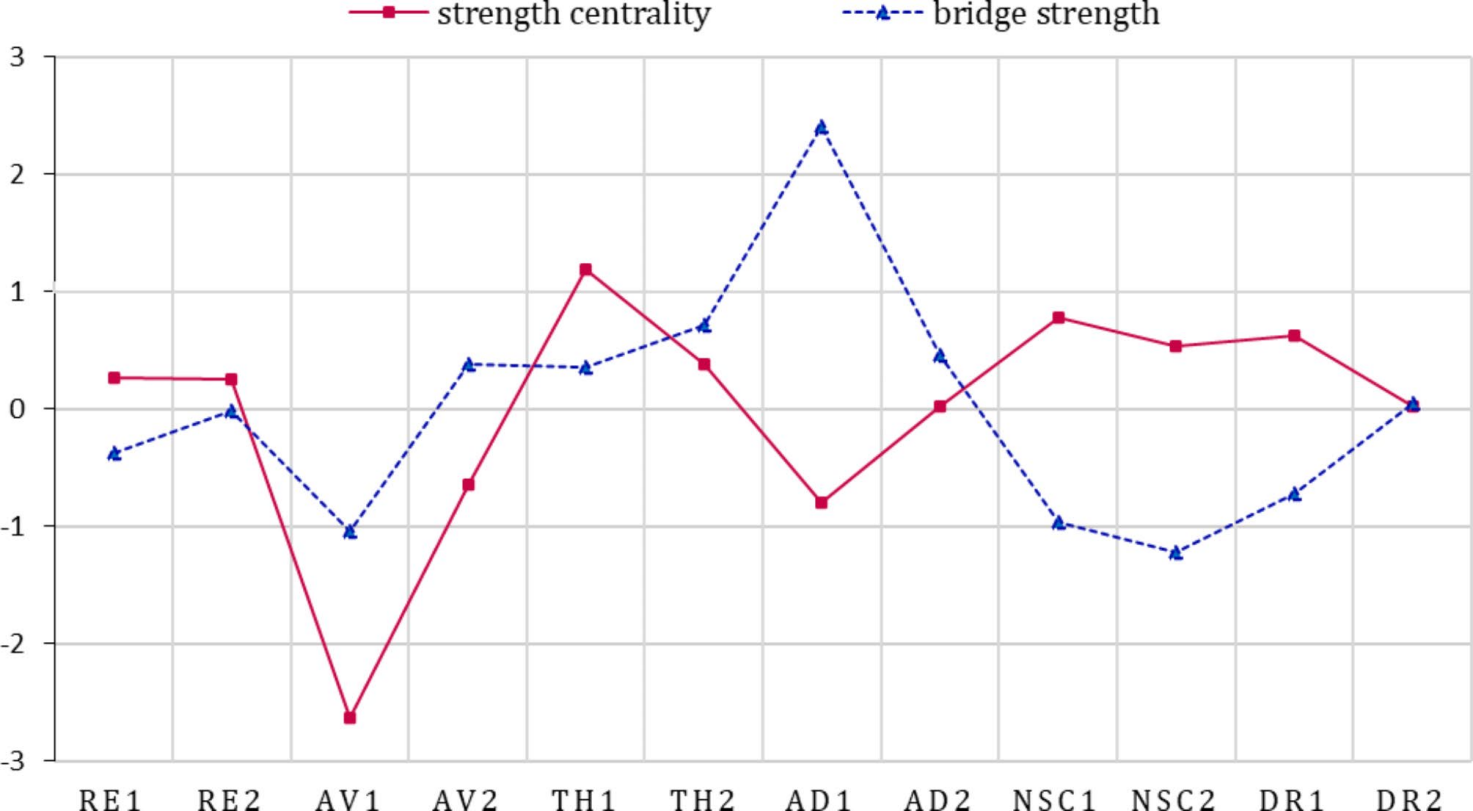
Standardized node strength centrality and bridge strength of CPTSD symptoms. *Notes*: RE1: nightmares; RE2: flashbacks; AV1: internal avoidance; AV2: external avoidance; TH1: hypervigilance; TH2: exaggerated startle response; AD1: long-term upset; AD2: emotional numbing; NSC1: feelings of failure; NSC2: feelings of worthlessness; DR1: feeling distant or cut off from others; DR2: difficulties feeling close to others

The results of strength centrality with normalization are shown as solid lines in Fig. 3. TH1 (hypervigilance) and NSC1 (feelings of failure), respectively belonging to PTSD and DSO, had the highest strength centrality. The calculation results of the bridge strength are shown by the dotted line in Fig. 3. The bridge strength of AD1 (long-term upset) from DSO was high.

The bootstrapping results of the edge weight confidence intervals are shown in Figure S1. The red line represents each edge weight value, and the gray areas on both sides represent the 95% confidence interval. The results showed that the confidence interval near the edge weights was small, especially for those edges with large weights. This indicated that the accuracy of the network estimation was at a high level.

The subset bootstrapping results of centrality are shown in Figure S3. The curve in the figure declines slowly, and the centrality values ​​of the subset and the original data remain highly correlated even after removing a large number of subjects, which means that the centrality estimate can be considered stable. The CS-coefficient of strength centrality was 0.75, and the CS-coefficient of bridge strength was 0.67, which indicate that the results of centrality have strong stability.

The difference test results of edge weights are shown in Figure S3. The small black boxes represent significant differences between the corresponding two edge weights. The difference test results of the strength centrality are shown in Figure S4. The black box indicates that there is a significant difference in the strength centrality between the corresponding two nodes. The values ​​of high centrality symptoms were statistically greater than most of the other symptoms.

The results of network structure, strength centrality and bridge strength in participants who experienced 2 or more trauma types were similar to those in 1368 participants, and the results are shown in the supplemental materials (see Figures S5 and S6).

### Bayesian network

**Fig. 4:**
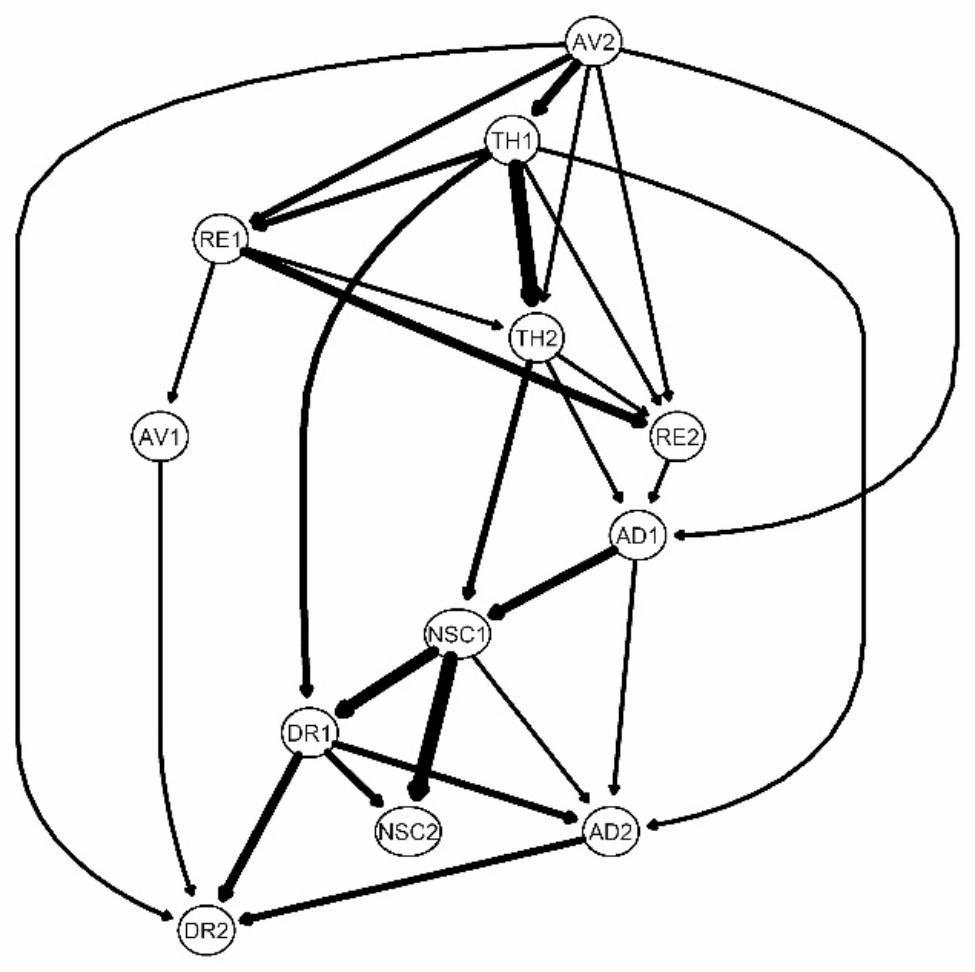
Directed acyclic graph of CPTSD symptoms. *Notes*: RE1: nightmares; RE2: flashbacks; AV1: internal avoidance; AV2: external avoidance; TH1: hypervigilance; TH2: exaggerated startle response; AD1: long-term upset; AD2: emotional numbing; NSC1: feelings of failure; NSC2: feelings of worthlessness; DR1: feeling distant or cut off from others; DR2: difficulties feeling close to others

Figure 4 shows the Bayesian network obtained by averaging the results of multiple bootstrapped samples. The upstream status of AV2 (external avoidance) and TH1 (hypervigilance) was evident, while difficulties feeling close to others (DR2) and emotional numbing (AD2) were downstream symptoms, relatively dependent on other symptoms in the network. Different from TH1, which was not only a highly central symptom but also an upstream symptom, NSC1 was a symptom with high centrality but a downstream position in the symptom flow. As demonstrated in the Bayesian network, it could be supposed that the high centrality of TH1 might be associated with its significant role in activating other CPTSD symptoms, whereas the high centrality of NSC1 indicated that it could be easily activated by many other CPTSD symptoms.

## Discussion

This study provides new evidence for the network structure of ICD-11 CPTSD in Chinese culture. Through a regularized partial correlation network, feelings of failure and hypervigilance were found to be the most central symptoms in the current study, and long-term upset was found to be the bridge symptom between the symptoms of PTSD and DSO. The results of the Bayesian network showed that hypervigilance was located upstream, while feelings of failure was located downstream, which indicated that hypervigilance may be predictive of other symptoms and that feelings of failure may be influenced by other symptoms.

The results of CFA showed that a six-factor correlated model of the ITQ fit best in our sample. A two-factor second-order model also fit well. These findings were consistent with previous studies, which indicate that the correlated six-factor first-order model was the best fitting model among community studies [7]. These results indicated that the ITQ is a valid measurement for CPTSD symptoms in China.

Results of network analysis showed that hypervigilance was one of the symptoms with high centrality in the CPTSD network. The Bayesian network further showed that hypervigilance was located upstream among CPTSD symptoms. Taken together, hypervigilance might be a core symptom in CPTSD. This could be explained by the cognitive model of PTSD symptom maintenance [37]. After exposure to a traumatic event, only those who gain a persistent sense of threat will develop PTSD. This view emphasizes that the occurrence of PTSD depends on a person’s assessment of trauma; when people believe that there are dangers all around them and everything is threatening, they enter a state of restlessness and vigilance. Even some tiny clues could provoke them into startle reactions. Individuals who suffer from hypervigilance symptoms after prolonged trauma (corresponding to the source of CPTSD) should be the focus of intervention because they are most likely to develop CPTSD. In the diagnosis of CPTSD, hypervigilance also requires greater attention because it implies the underlying developing progress of follow-up symptoms.

Centrality estimation also showed that feeling of failure was another high central symptom in the CPTSD network. The finding of this study was broadly consistent with the existing empirical evidence that negative self-concept (especially feeling of worthlessness) possesses high centrality [14–16]. However, the Bayesian network presented feeling of failure as a downstream symptom. This suggests that the reason why feeling of failure has abundant associations with other symptoms is that it could be easily predicted by other symptoms. According to Maercker and colleagues, this phenomenon usually occurs when other symptoms cause functional impairment [38, 39].

The results of the present study showed that long-term upset from DSO was symptoms of high bridge strength between PTSD and DSO. Our network results found that the long-term upset, was clearly linked to the PTSD symptom clusters. A DSM-5-based PTSD network analysis study found that anhedonia and dysphoria are central symptoms of PTSD, indicating that emotional dysregulation can have an important impact on PTSD symptom clusters [40]. Therefore, long-term upset might bridge the symptoms of PTSD and DSO through emotional dysregulation. A study on CPTSD treatment also identified long-term upset as an important mediator between DSO and PTSD, advocating adaptive emotion regulation strategies in treatment [41].

There are limitations that are relevant to studies capturing the CPTSD symptom network that deserve mention in the context of this analysis. First, assessing CPTSD symptoms relies on self-reporting (via ITQ in this study). Although ITQ is proven to be an effective tool for measuring CPTSD [20], reporting bias still exists. More research based on structured clinical interviews is needed. Second, we attempted to ensure the representativeness of the sample among Chinese young adults. However, conclusions should be generalized to adults of other age groups with caution. Third, we used a Bayesian network to provide potential causality between symptom associations; however, the Bayesian network was based on a directed acyclic graph, which did not consider bidirectional influence or feedback loops [11]. Network analysis based on longitudinal data is needed to further understand the causal and temporal relationships between CPTSD symptoms. Finally, the composition of our sample had a gender skew, with more participants being women. Future studies should consider the sex ratio when recruiting participants.

Despite these limitations, the current study provides the first insight into the network structure of CPTSD in young Chinese adults. We identified that hypervigilance is a central symptom and may be quite predictive of other symptoms of CPTSD. In contrast, feelings of failure is a highly central symptom, yet it may be influenced by other symptoms. The prevalence of CPTSD was higher than that of PTSD in our sample, which is consistent with previous studies [42]. These results indicated that PTSD symptoms are often accompanied by DSO symptoms in people with a history of trauma, which demonstrated the importance of the WHO treating CPTSD as an independent diagnosis in the ICD-11. In the current study, we further found that PTSD and DSO clusters may be linked through long-term upset because long-term upset established links between PTSD and DSO symptom clusters. Our results have clinical implications, indicating that patients who experience long-term trauma and exhibit hypervigilance should be the focus of clinical intervention in CPTSD. Moreover, hypervigilance and long-term upset should also be given more attention when diagnosing CPTSD.

### Electronic supplementary material

Below is the link to the electronic supplementary material.

**Supplementary Material 1**: Additional Figures. **Figure S1**. Bootstrapped confidence intervals (CIs) of the edge weights of the CPTSD symptom network. **Figure S2**. Subsetting bootstrap for the CPTSD network. **Figure S3**. Edge weight difference tests for the CPTSD symptom network. **Figure S4**. Node strength centrality difference tests for the CPTSD symptom network. **Figure S5**. Regularized partial correlation network of CPTSD symptoms among participants who experienced 2 or more trauma types. **Figure S6**. Standardized node strength centrality and bridge strength of CPTSD symptoms among participants who experienced 2 or more trauma types

## Data Availability

The dataset generated and/or analyzed during the current study are not publicly available due to ethic issues involving participant’s data and privacy but are available from the corresponding author on reasonable request.

## References

[1] World Health Organization. International Classification of Diseases-11the Version (ICD-11). 2018. Retrieved Dec 23 2021 from [https://icd.who.int/browse11/l-m/en#/http://id.who.int/icd/entity/585833559].

[2] Herman JL, Complex PTSD (1992). A syndrome in survivors of prolonged and repeated trauma. J Trauma Stress.

[3] Hyland P, Murphy J, Shevlin M, Vallières F, McElroy E, Elklit A (2017). Variation in post-traumatic response: the role of trauma type in predicting ICD-11 PTSD and CPTSD symptoms. Soc Psychiatry Psychiatr Epidemiol.

[4] Ben-Ezra M, Karatzias T, Hyland P, Brewin CR, Cloitre M, Bisson JI (2018). Posttraumatic stress disorder (PTSD) and complex PTSD (CPTSD) as per ICD‐11 proposals: a population study in Israel. Depress Anxiety.

[5] Gilbar O, Hyland P, Cloitre M, Dekel R (2018). ICD-11 complex PTSD among Israeli male perpetrators of intimate partner Violence: construct validity and risk factors. J Anxiety Disord.

[6] Hansen M, Hyland P, Karstoft KI, Vaegter HB, Bramsen RH, Nielsen AB (2017). Does size really matter? A multisite study assessing the latent structure of the proposed ICD-11 and DSM-5 diagnostic criteria for PTSD. Eur J Psychotraumatol.

[7] Redican E, Nolan E, Hyland P, Cloitre M, McBride O, Karatzias T (2021). A systematic literature review of factor analytic and mixture models of ICD-11 PTSD and CPTSD using the International Trauma Questionnaire. J Anxiety Disord.

[8] Fried EI, Cramer AO (2017). Moving forward: challenges and directions for psychopathological network theory and methodology. Perspect Psychol Sci.

[9] Liang Y, Li F, Zhou Y, Liu Z (2021). Evolution of the network pattern of posttraumatic stress symptoms among children and adolescents exposed to a Disaster. J Anxiety Disord.

[10] Borsboom D, Cramer AO (2013). Network analysis: an integrative approach to the structure of psychopathology. Annu Rev Clin Psychol.

[11] McNally RJ (2016). Can network analysis transform psychopathology?. Behav Res Ther.

[12] Jones PJ, Ma R, McNally RJ (2021). Bridge centrality: a network approach to understanding comorbidity. Multivar Behav Res.

[13] Karatzias T, Shevlin M, Hyland P, Ben-Ezra M, Cloitre M, Owkzarek M (2020). The network structure of ICD‐11 complex post‐traumatic stress disorder across different traumatic life events. World Psychiatry.

[14] Knefel M, Karatzias T, Ben-Ezra M, Cloitre M, Lueger-Schuster B, Maercker A (2019). The replicability of ICD-11 complex post-traumatic stress disorder symptom networks in adults. Br J Psychiatry.

[15] Knefel M, Lueger-Schuster B, Bisson J, Karatzias T, Kazlauskas E, Roberts NP (2020). A cross‐cultural comparison of icd‐11 complex posttraumatic stress disorder symptom networks in Austria, the United Kingdom, and Lithuania. J Trauma Stress.

[16] Levin Y, Hyland P, Karatzias T, Shevlin M, Bachem R, Maercker A (2021). Comparing the network structure of ICD-11 PTSD and complex PTSD in three African countries. J Psychiatr Res.

[17] McElroy E, Shevlin M, Murphy S, Roberts B, Makhashvili N, Javakhishvili J (2019). ICD-11 PTSD and complex PTSD: structural validation using network analysis. World Psychiatry.

[18] Schiess-Jokanovic J, Knefel M, Kantor V, Weindl D, Schäfer I, Lueger-Schuster B (2022). The boundaries between complex posttraumatic stress disorder symptom clusters and post-migration living difficulties in traumatised Afghan refugees: a network analysis. Conf Health.

[19] Weathers FW, Litz BT, Keane TM, Palmieri PA, Marx BP, Schnurr PP. The life events Checklist for DSM-5 (LEC-5). Instrument available from the National Center for PTSD at [www.ptsd.va.gov]; 2013.

[20] Cloitre M, Shevlin M, Brewin CR, Bisson JI, Roberts NP, Maercker A (2018). The International Trauma Questionnaire: development of a self-report measure of ICD-11 PTSD and complex PTSD. Acta Psychiatr Scand.

[21] Ho GW, Karatzias T, Cloitre M, Chan AC, Bressington D, Chien WT (2019). Translation and validation of the Chinese ICD-11 international trauma questionnaire (ITQ) for the assessment of posttraumatic stress disorder (PTSD) and complex PTSD (CPTSD). Eur J Psychotraumatol.

[22] Muthén LK, Muthén BO (2013). Mplus user’s guide.

[23] Bentler PM (1990). Comparative fit indexes in structural models. Psychol Bull.

[24] Tucker LR, Lewis C (1973). A reliability coefficient for maximum likelihood factor analysis. Psychometrika.

[25] Steiger JH (1990). Structural model evaluation and modification: an interval estimation approach. Multivar Behav Res.

[26] Chen FF (2007). Sensitivity of goodness of fit indexes to lack of measurement invariance. Struct Equ Model.

[27] Epskamp S, Fried EI (2018). A tutorial on regularized partial correlation networks. Psychol Methods.

[28] Epskamp S, Cramer AO, Waldorp LJ, Schmittmann VD, Borsboom D, Qgraph (2012). Network visualizations of relationships in psychometric data. J Stat Softw.

[29] Friedman J, Hastie T, Tibshirani R (2008). Sparse inverse covariance estimation with the graphical lasso. Biostatistics.

[30] Bringmann LF, Elmer T, Epskamp S, Krause RW, Schoch D, Wichers M (2019). What do centrality measures measure in psychological networks?. J Abnorm Psychol.

[31] Opsahl T, Agneessens F, Skvoretz J (2010). Node centrality in weighted networks: generalizing degree and shortest paths. Soc Networks.

[32] Epskamp S, Fried EI. Package ‘bootnet’. R package version. 2020;1.

[33] Epskamp S, Borsboom D, Fried EI (2018). Estimating psychological networks and their accuracy: a tutorial paper. Behav Res Methods.

[34] Daly R, Shen Q. Methods to accelerate the learning of bayesian network structures. Paper presented at the Proceedings of the 2007 UK Workshop on Computational Intelligence, London: Imperial College; 2007.

[35] Scutari M (2010). Learning bayesian networks with the bnlearn R package. J Stat Softw.

[36] McNally R, Mair P, Mugno B, Riemann B (2017). Co-morbid obsessive–compulsive disorder and depression: a bayesian network approach. Psychol Med.

[37] Lancaster SL, Rodriguez BF, Weston R (2011). Path analytic examination of a cognitive model of PTSD. Behav Res Ther.

[38] Maercker A, Brewin CR, Bryant RA, Cloitre M, Reed GM, Van Ommeren M (2013). Proposals for mental disorders specifically associated with stress in the International classification of Diseases-11. Lancet.

[39] Maercker A, Brewin CR, Bryant RA, Cloitre M, van Ommeren M, Jones LM (2013). Diagnosis and classification of disorders specifically associated with stress: proposals for ICD-11. World Psychiatry.

[40] Benfer N, Bardeen JR, Cero I, Kramer LB, Whiteman SE, Rogers TA (2018). Network models of posttraumatic stress symptoms across trauma types. J Anxiety Disord.

[41] Karatzias T, Shevlin M, Hyland P, Brewin CR, Cloitre M, Bradley A (2018). The role of negative cognitions, emotion regulation strategies, and attachment style in complex post-traumatic stress disorder: implications for new and existing therapies. Br J Clin Psychol.

[42] Tian Y, Wu X, Wang W, Zhang D, Yu Q, Zhao X (2020). Complex posttraumatic stress disorder in Chinese young adults using the International Trauma Questionnaire (ITQ): a latent profile analysis. J Affect Disord.

