# Supplementary material for: Symptom structure of complex posttraumatic stress disorder among Chinese young adults with childhood trauma: a network analysis
Source: BMC Psychiatry. 2023 Dec 5;23:911. doi: 10.1186/s12888-023-05423-2 (PMC10698995; doi:10.1186/s12888-023-05423-2)

**Figure S1.** Bootstrapped confidence intervals (CIs) of the edge weights of the CPTSD symptom network. The red line indicates the edge weight values, and the gray area indicates the 95% CIs.


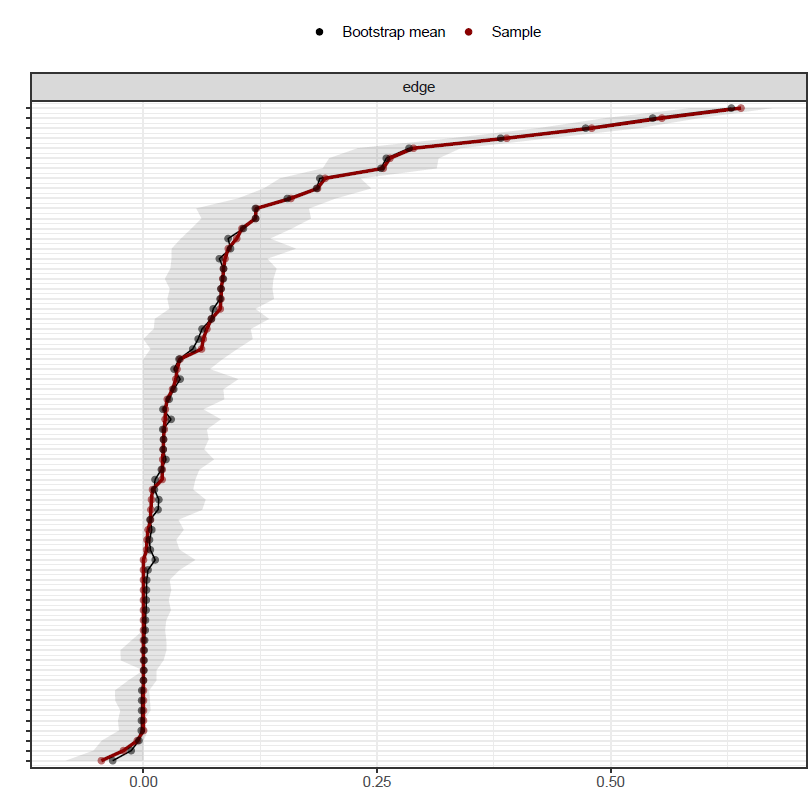


**Figure S2.** Subsetting bootstrap for the CPTSD network. Figures show the average correlations between centrality indices of the original sample and subsamples with fewer participants.


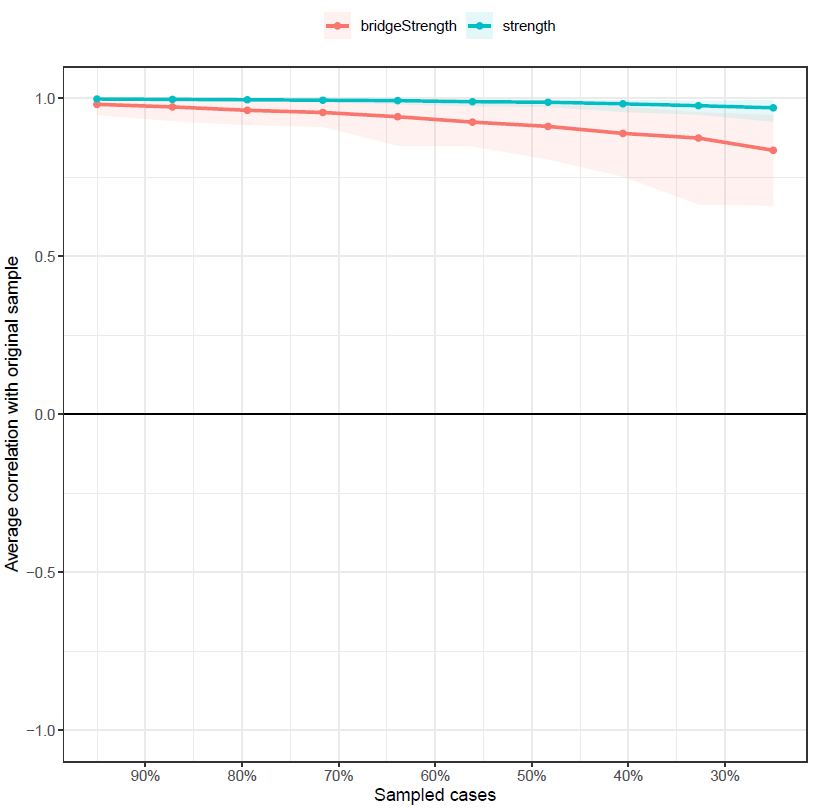


**Figure S3.** Edge weight difference tests for the CPTSD symptom network. The black boxes represent an edge that differs significantly from another edge (α = 0.05).

Notes: RE1: nightmares; RE2: flashbacks; AV1: internal avoidance; AV2: external avoidance; TH1: hypervigilance; TH2: exaggerated startle response; AD1: long-term upset; AD2: emotional numbing; NSC1: feelings of failure; NSC2: feelings of worthlessness; DR1: feeling distant or cut off from others; DR2: difficulties feeling close to others.


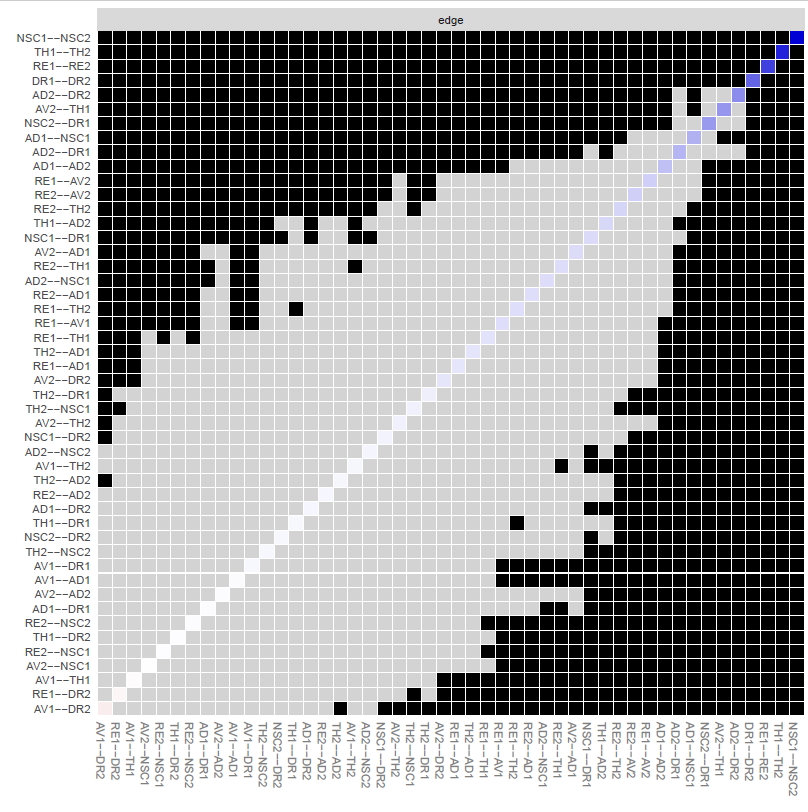


**Figure S4.** Node strength centrality difference tests for the CPTSD symptom network. The black boxes represent a node that differs significantly from another node (α = 0.05).

Notes: RE1: nightmares; RE2: flashbacks; AV1: internal avoidance; AV2: external avoidance; TH1: hypervigilance; TH2: exaggerated startle response; AD1: long-term upset; AD2: emotional numbing; NSC1: feelings of failure; NSC2: feelings of worthlessness; DR1: feeling distant or cut off from others; DR2: difficulties feeling close to others.


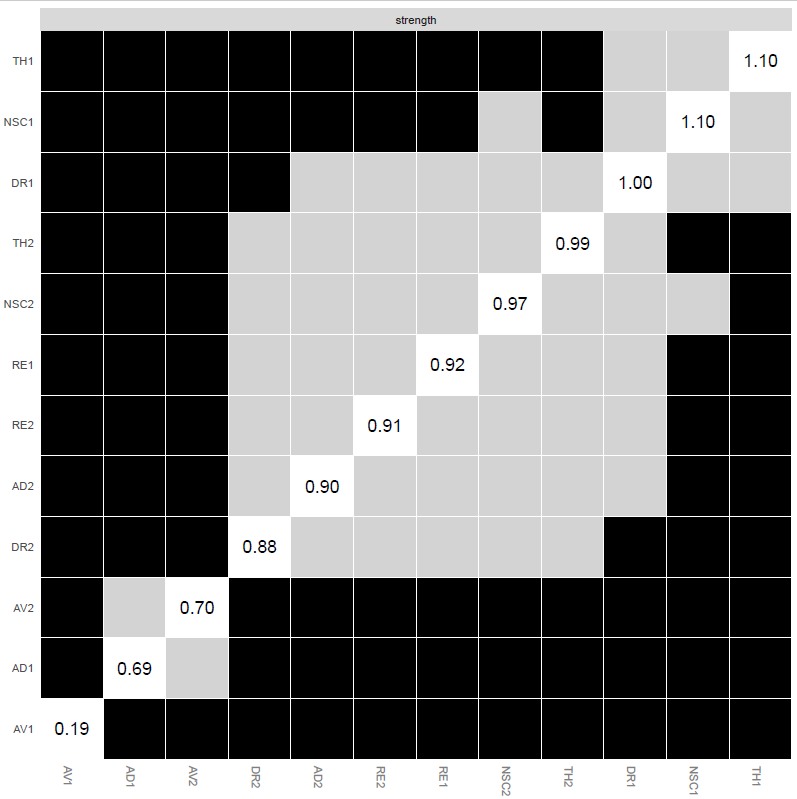


**Figure S5**. Regularized partial correlation network of CPTSD symptoms among participants who experienced 2 or more trauma types

Notes: RE1: nightmares; RE2: flashbacks; AV1: internal avoidance; AV2: external avoidance; TH1: hypervigilance; TH2: exaggerated startle response; AD1: long-term upset; AD2: emotional numbing; NSC1: feelings of failure; NSC2: feelings of worthlessness; DR1: feeling distant or cut off from others; DR2: difficulties feeling close to others


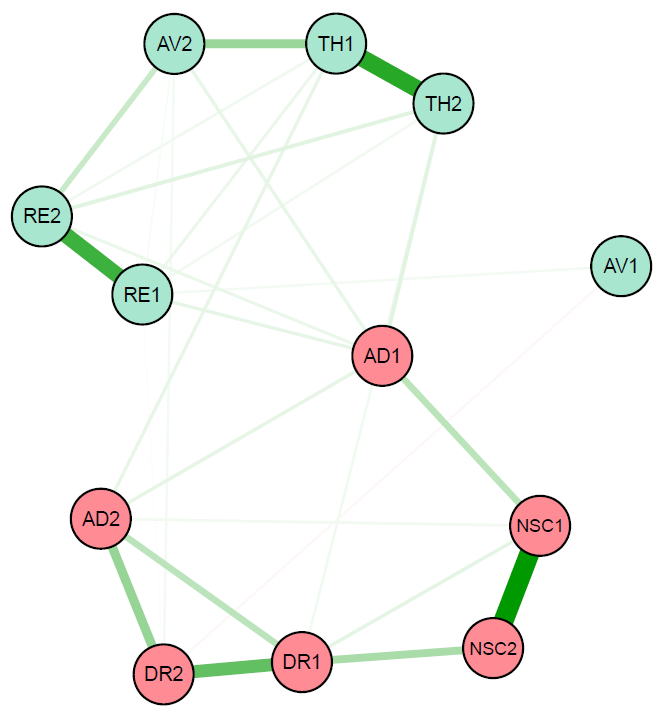


**Figure S6.** Standardized node strength centrality and bridge strength of CPTSD symptoms among participants who experienced 2 or more trauma types

Notes: RE1: nightmares; RE2: flashbacks; AV1: internal avoidance; AV2: external avoidance; TH1: hypervigilance; TH2: exaggerated startle response; AD1: long-term upset; AD2: emotional numbing; NSC1: feelings of failure; NSC2: feelings of worthlessness; DR1: feeling distant or cut off from others; DR2: difficulties feeling close to others


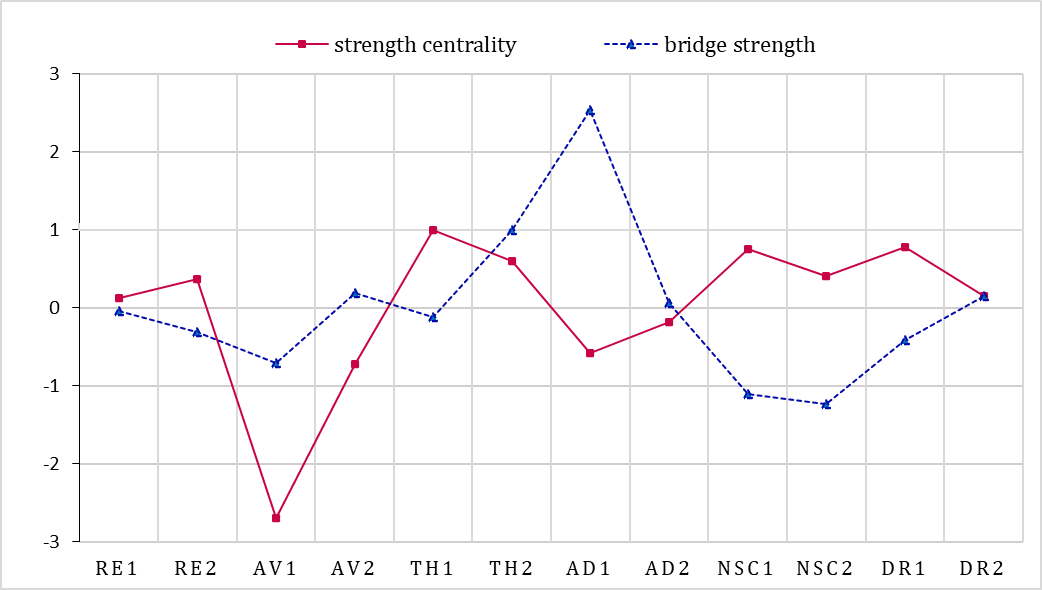

Supplement: Supplementary file 1 — Supplementary Material 1: Additional Figures. Figure S1. Bootstrapped confidence intervals (CIs) of the edge weights of the CPTSD symptom network. Figure S2. Subsetting bootstrap for the CPTSD network. Figure S3. Edge weight difference tests for the CPTSD symptom network. Figure S4. Node strength centrality difference tests for the CPTSD symptom network. Figure S5. Regularized partial correlation network of CPTSD symptoms among participants who experienced 2 or more trauma types. Figure S6. Standardized node strength centrality and bridge strength of CPTSD symptoms among participants who experienced 2 or more trauma types [file 12888_2023_5423_MOESM1_ESM.docx]
